# Navigation-based endoscopic enucleation (NBEE) of large mandibular cystic lesions involving the ramus

**DOI:** 10.1186/s12903-024-03859-w

**Published:** 2024-01-16

**Authors:** Yan Wang, Xiaoxian Xu, Zixian Huang, Yongkang Cai, Yilin He, Songling Fang, Bo He, Zhiquan Huang

**Affiliations:** 1 Department of Oral and Maxillofacial Surgery, Sun Yat-sen Memorial Hospital, Sun Yat-sen University, 107th Yanjiang Xi Road, Guangzhou, Guangdong 510120 China; 2 Department of Anesthesiology, Sun Yat-sen Memorial Hospital, Sun Yat-sen University, 107th Yanjiang Xi Road, Guangzhou, Guangdong 510120 China

**Keywords:** Large mandibular cystic lesion, Mandibular ramus, Endoscopy, Surgical navigation

## Abstract

**Objective:**

The aim of this study was to present an innovative surgical protocol, navigation-based endoscopic enucleation (NBEE) for the treatment of large mandibular cystic lesions involving the mandibular ramus.

**Methods:**

Twelve patients who presented with a large mandibular cystic lesion involving the mandibular ramus were enrolled in this study. Preoperative planning and intraoperative navigation were performed in all 12 patients.

**Results:**

All patients in this study were treated with navigation-based endoscopic enucleation successfully. The follow-up period ranged from 7 to 10 months. Bone regenerated was found in all patients postoperatively. Three patients experienced temporary mandibular nerve palsy, and all relieved within 2 months. No pathological bone fracture was found during surgery.

**Conclusions:**

The use of navigation-based endoscopic enucleation (NBEE) for the treatment of large mandibular cystic lesions involving the ramus proved to be an effective method for complete and precise enucleation of the cystic lesion that also preserved the surrounding tissue.

## Introduction

The mandibles are prone to a wide variety of cystic lesions, odontogenic and nonodontogenic, and reactive and neoplastic. Most of them are slow growing and asymptomatic or have mild symptoms. Lesions may grow large in size and damage surrounding structures, causing facial swelling, infection, root resorption, nerve injuries, pathological bone fractures or even malocclusion [1, 2]. Treatment methods range from conservative to radical. Currently, enucleation and decompression are the two main surgical procedures to treat mandibular cystic lesions [3, 4]. For small lesions, primary enucleation with or without allogenic bone grafting is the most common choice [5–7]. However, enucleation of large lesions may pose an increased risk of nerve bundle injuries and bone fractures, as well as residual lesions, especially when the lesion involves the mandibular ramus [8]. Decompression or marsupialization is a less invasive alternative method for treating large lesions, but this approach still has its drawbacks, including prolonged treatment period, discomfort caused by obturators and decompression tubes, more than half of the cases need a secondary enucleation, and remnants of the epithelial lining may lead to recurrence [9]. Radical procedures, including segmental/marginal resection of the involved mandible with or without bone flap reconstruction, are mainly applied for the treatment of large odontogenic neoplastic cystic lesions, such as ameloblastoma. This can reduce the recurrence rate but produce deformity of the jaws and influence the patients’ quality of life [10, 11]. 

Endoscopy has the advantages of excellent visibility, better illumination and sufficient magnification and has been successfully applied in various intraoral surgeries, including jawbone cyst enucleation [12–15]. Our team previously reported the use of endoscopic-assisted enucleation for the treatment of large mandibular odontogenic cysts, and the study showed that compared with traditional enucleation, endoscopic enucleation proved to be a more effective procedure as it provided complete enucleation of the lesion and preserved the surrounding tissue [16]. However, endoscopic assistance alone also has its own limitations: the recognition of important tissue, such as nerves and residual lesions, is still subjective and mainly depends on the surgeon’s clinical experience; most of the cystic cavities’ three-dimensional structures are irregular, and some of them are even multilocular, so surgeons cannot reach every possible corner of the cavity when performing endoscopy alone; and some impacted teeth in the cavity are difficult to locate via endoscopy.

Surgical navigation has been increasingly applied in oral and craniomaxillofacial surgery over the last 20 years. It provides surgeons with real-time visualization of the position and direction of the surgical instruments in relation to the patient’s anatomy and allows surgeons obtain clear and objective anatomic identification during surgery. Preoperative computer simulation combined with intraoperative navigation has been proven to be an effective treatment modality for improving surgical outcomes [17]. However, few studies have focused on the application of surgical navigation technology in combination with endoscopic surgery for the treatment of large mandibular cystic lesions. The aim of this study was to present an innovative surgical protocol, navigation-based endoscopic enucleation (NBEE), which integrates intraoperative surgical navigation technology and endoscopic-assisted enucleation techniques, for the treatment of large mandibular cystic lesions involving the mandibular ramus.

## Materials and methods

### Patients

This study was approved by the Institutional Ethics Committee of Sun Yat-sen Memorial Hospital (Approval No. SYSJS-2023-048-01) and followed the Helsinki Declaration. Written informed consent was obtained from all patients. A total of 12 consecutive patients who presented with a large mandibular cystic lesion involving the mandibular ramus and were admitted to the Department of Oral and Maxillofacial Surgery, Sun Yat-sen Memorial Hospital, Sun Yat-sen University, between July 2022 and October 2022 were enrolled in this study. The 12 patients (7 males and 5 females) had an average age of 30.6 years (range, 12–54 years). The preoperative diagnosis of all patients was confirmed by facial spiral computed tomography (CT) scan and orthopantomography before surgery. The involved teeth in the cystic cavity of all patients were treated with root canal therapy preoperatively.

Inclusion criteria were as follows:

1. Radiologic diagnosis of unilateral mandibular cystic lesions involving the mandibular ramus.
2. Radiologic diameter of the lesion ≥ 2 cm.
3. Lesion recurred after ineffective treatment with decompression/marsupialization.

The exclusion criteria were as follows:

1. Radiologic diagnosis of a solid tumor of the jaw.
2. Lesion recurred after failed enucleation or resection.
3. Radiologic diameter of the cyst < 2 cm.

### Preoperative preparation

#### Image segmentation of the lesion and surrounding structures

**Fig. 1:**
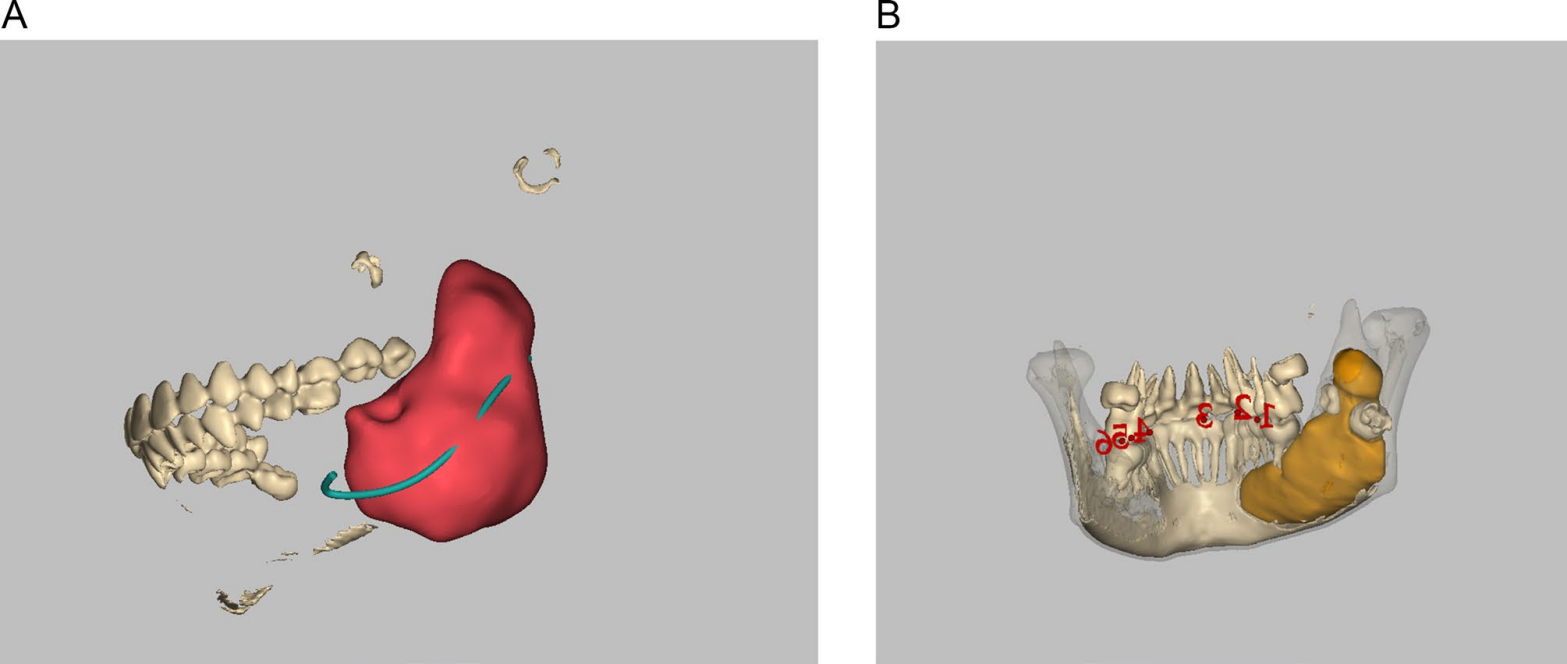
(**A**) Segmentation of the lesion and inferior alveolar nerve by Mimics 20.0. (**B**) Segmentation of the lesion and impacted teeth by ProPlan CMF 3.0

The preoperative CT file of the patient was elaborated into DICOM (Digital Imaging and Communications in Medicine) format and imported into surgical planning software for image segmentation (Mimics 20.0 or ProPlan CMF 3.0). The segmentation process was performed by the following two steps: (1) we segmented the contours of the cystic lesion, then we created a 3D reconstruction of the segmented lesion with different colors to clearly display the location and extent of the whole cystic lesion; (2) we segmented the inferior alveolar nerve and/or impact tooth separately according to the treatment purpose (Fig. 1).

#### Reference point design

**Fig. 2:**
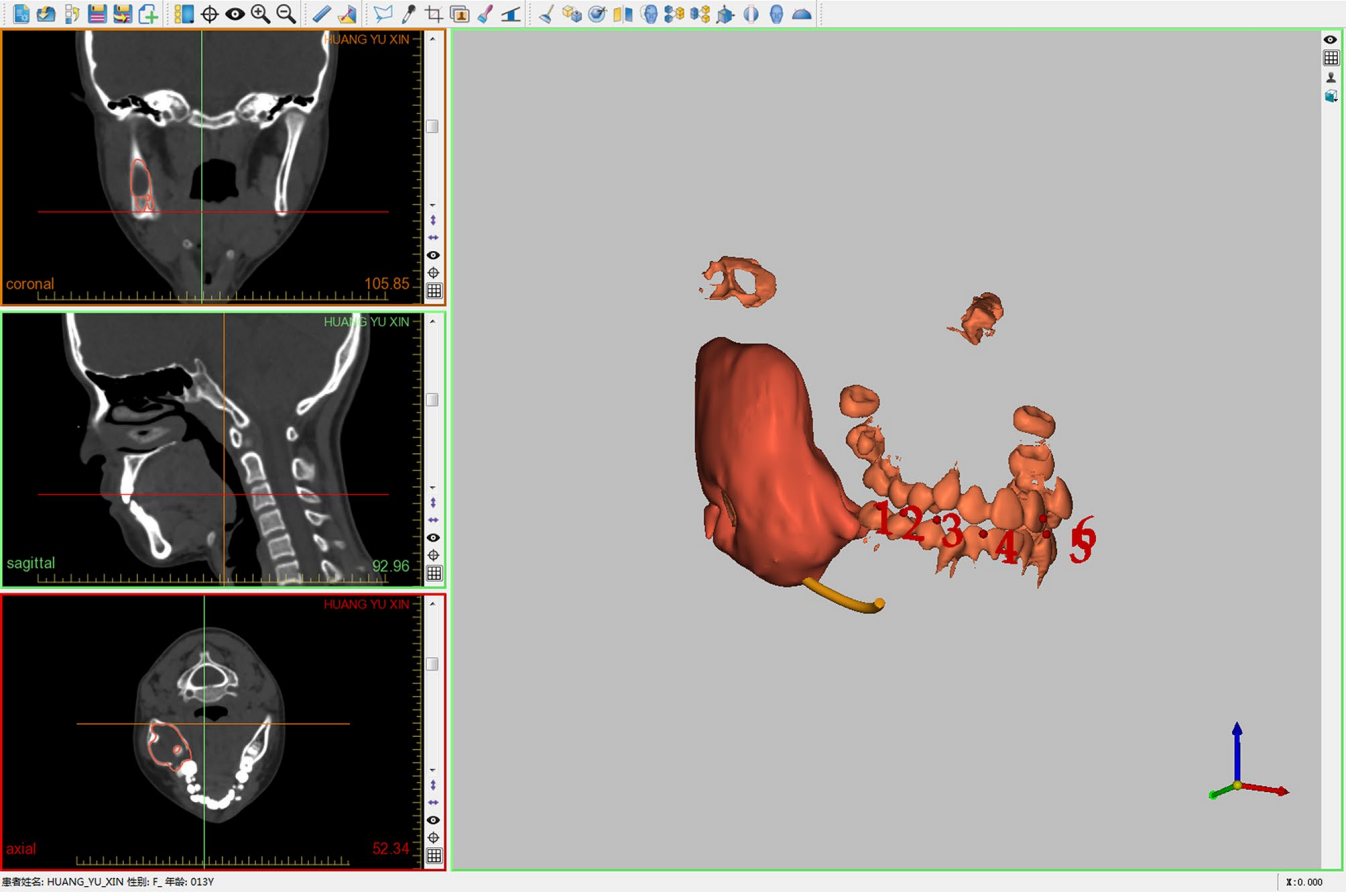
Image segmentation was imported into AccuNavi-A 2.0, and registration points were designed

The segmentation images were then converted into STL (Standard Triangulation Language) format and imported into navigation software (AccuNavi-A 2.0, Shanghai, China). We applied the point registration method for intraoperative registration by designing anatomical landmarks on mandibular teeth (at least 5 teeth for each patient) (Fig. 2).

### Intraoperative navigation and surgical process

#### Intraoperative registration

**Fig. 3:**
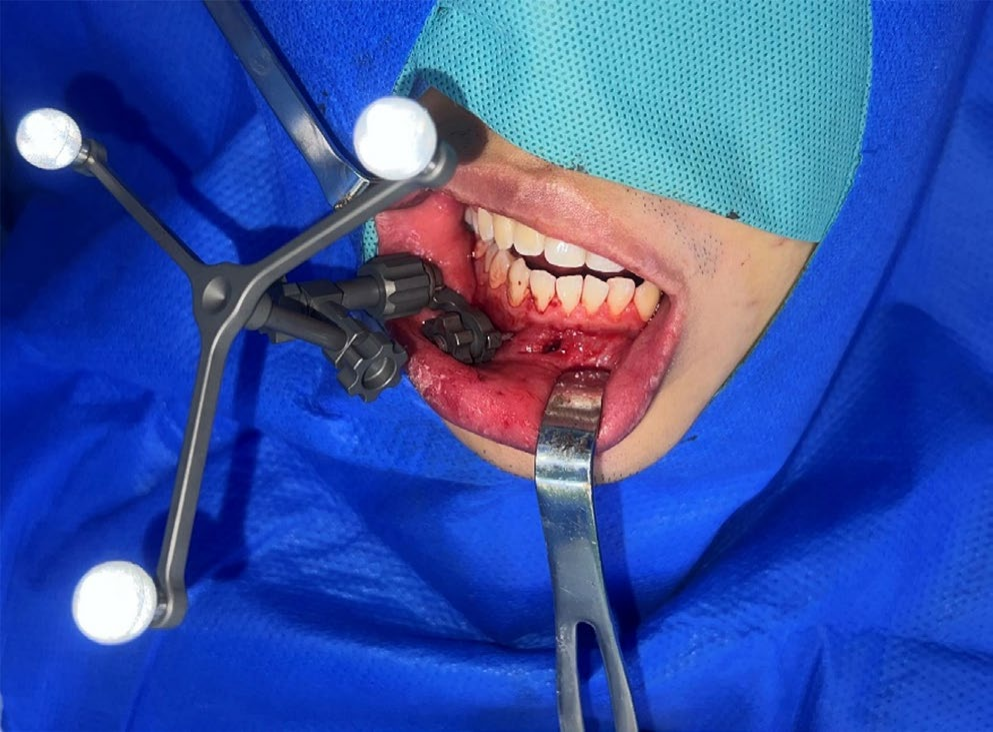
A mandible-specialized digital reference frame (DRF) was rigidly fixed on the patient’s contralateral mandibular alveolar bone

Intraoperative registration was performed on patients under general anesthesia with nasoendotracheal intubation. An oral and maxillofacial surgery specialized navigation system (AccuNavi-A Surgical Navigation System, Shanghai, China) was used in all cases, and frameless stereotaxy was applied during navigation. The steps were as follows: (1) we used infrared cameras to track the navigation pointer and trackers; (2) the patient’s position was identified using a mandible-specialized digital reference frame (DRF), which was rigidly fixed on the patient’s contralateral mandibular alveolar bone; and (3) registration was then accomplished by performing the point registration method, which was designed on the mandibular teeth preoperatively. Registration accuracy was tested visually by repeatedly pinpointing. A margin of error of < 1.0 mm was considered acceptable during the surgery (Fig. 3).

#### Surgical process

A buccal trapezoidal mucoperiosteal incision was made from the molar region to the anterior ascending ramus, depending on the lesion size. Subperiosteal blunt dissection was then performed to expose the bony surface.

After blunt dissection of the cystic lesion around the bony window, the navigation probe was used to evaluate and locate each wall of the cystic cavity and the route of the inferior alveolar nerve canal, as well as the impacted tooth if necessary.

Real-time endoscope-based navigation was then performed (Karl Storzendoskope system, Cat. No. 22,201,020). We fixed a clamped DRF on a 0-degree-angled 4 mm endoscope and inserted the scope into the appropriate hole (in our cases, 0-degree-angled use hole #7 and 70-degree-angled use hole #8) on a calibration block to finish the calibration procedure.

**Fig. 4:**
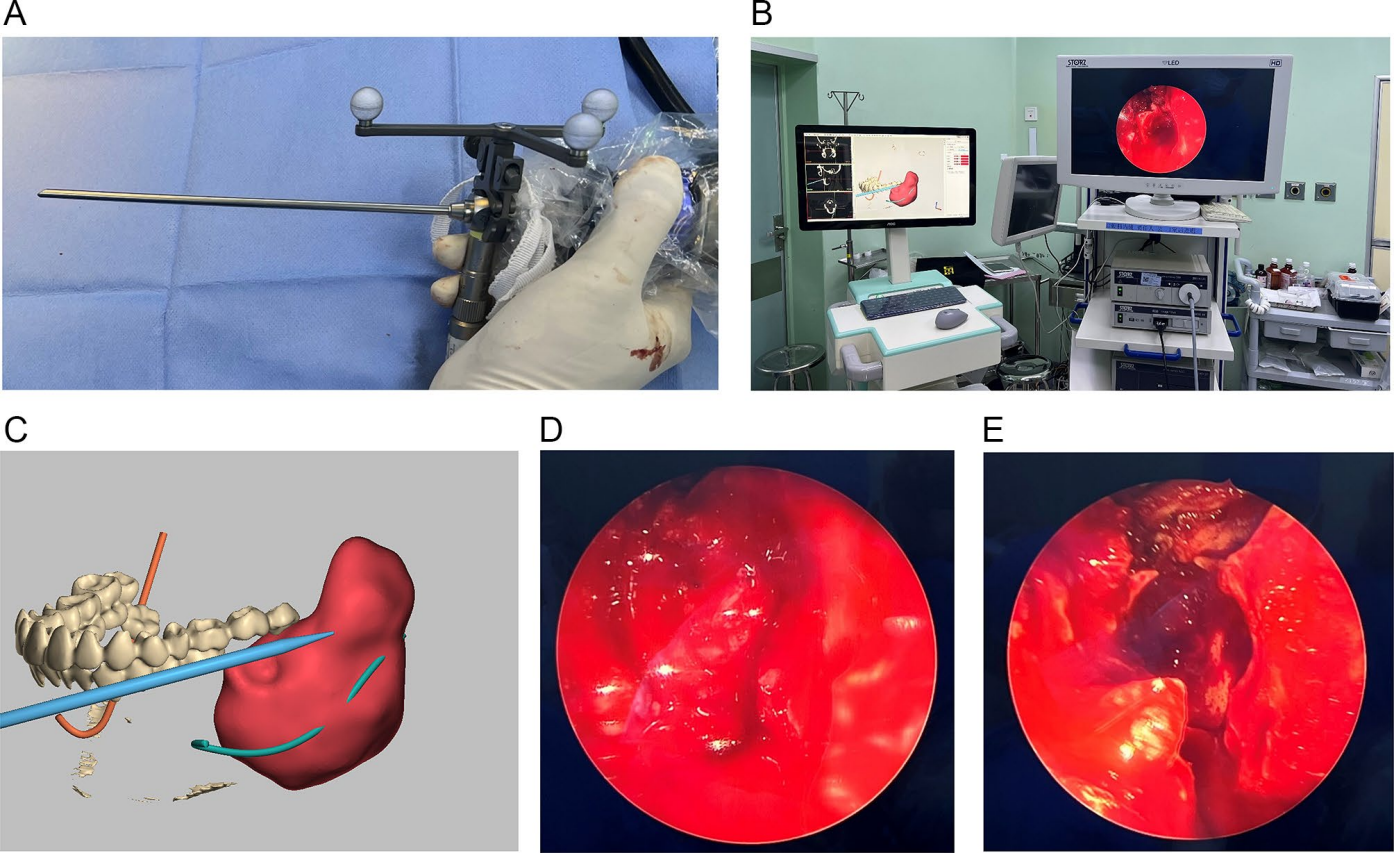
(**A**) Endoscope calibration using a DRF; (**B**) Joint guidance of navigation and endoscope during surgery; (**C**) A calibrated 70-degree-angled endoscope was introduced into the cystic cavity for evaluation; (**D**) The residual lesion was located and observed at the upper wall (at ramus area) of the cystic cavity; (**E**) An empty cystic bony wall of the mandibular angle area after complete curettage

After calibration, the lesion was curetted under the joint guidance of navigation and endoscopy. Exposed nerve bundles and impacted teeth were also clearly recognized during this procedure, and continuity of the nerve was carefully preserved. After the main lesion was removed, a 70-degree-angled endoscope was calibrated and introduced into the cystic cavity for cavity evaluation, especially for lesions located in the ramus region and root of the involved teeth. The bony cystic wall was completely curetted and residual lesions on the root were completely eradicated by using a curette and an electrical round burr, respectively. The empty cystic cavity was irrigated with saline solution. The incision site was sutured (Fig. 4).

## Results

All patients in this study were treated with navigation-based endoscopic enucleation. Preoperative planning and intraoperative navigation were performed successfully in all 12 patients. The mean operation time was 99.2 min (range, 65–125 min). The nerve bundle of 9 patients was exposed during surgery after main lesion removal, and all of them were visualized and recognized under navigation-based endoscopy. Five patients had impacted teeth, which were all precisely located and extracted under the joint guidance of navigation and endoscopy.

Postoperative pathological diagnosis revealed 6 odontogenic cysts, 1 odontogenic keratocyst, 4 ameloblastomas (unicystic) and 1 ameloblastoma (solid-cystic). All patients were followed for a minimum of 7 months postoperatively, with a mean of 8.8 months (range, 7–10 months). Bone regenerated was found in all patients via postoperative orthopantomography and CT scan. No serious complications occurred in any of the patients. Three patients experienced temporary mandibular nerve palsy (lower labial numbness), and all relieved within 2 months. No permanent nerve injury was observed. No pathological bone fracture was found during surgery. One patient had a postoperative operation site infection, which was subsequently managed by cavity irrigation (Table 1).

**Table 1:** Patient details and clinical characteristics

Pt. No.	Age (years)	Sex	Lesion side	Pathology	Previous treatment	Operation time(mins)	Temporary nerve palsy	Permanent nerve injury	Pathological bone fracture	Follow-up(months)	Recurrence
1	24	M	Left	Ameloblastoma(unicystic)	Marsupialization	125	Yes	No	No	10	No
2	41	M	Right	Odontogenic cyst	None	90	No	No	No	10	No
3	14	F	Right	Ameloblastoma(solid-cystic)	Marsupialization	115	Yes	No	No	10	No
4	37	M	Right	Odontogenic cyst	None	100	No	No	No	10	No
5	12	F	Right	Odontogenic cyst	None	115	No	No	No	9	No
6	42	F	Right	Odontogenic cyst	None	105	No	No	No	9	No
7	27	M	Left	Ameloblastoma(unicystic)	Marsupialization	122	Yes	No	No	9	No
8	45	M	Left	Ameloblastoma(unicystic)	None	65	No	No	No	8	No
9	17	M	Left	Odontogenic cyst	None	90	No	No	No	8	No
10	20	F	Left	Ameloblastoma(unicystic)	None	90	No	No	No	8	No
11	34	F	Right	Odontogenic keratocyst	None	85	No	No	No	7	No
12	54	M	Left	Odontogenic cyst	None	88	No	No	No	7	No

## Discussion

In the treatment of large mandibular cystic lesions involving the mandibular ramus, the biggest challenge has been eliminating the lesion to ensure no recurrence and few complications. In this study, for the first time, an intraoperative 3D navigation system-based endoscopic enucleation technology was used for exhaustive and safe surgery. The application of NBEE provided a clear and panoramic view of the cystic cavity via magnified high-resolution images and two different angled endoscopes; moreover, it reminded surgeons of the precise location of the images as well as the exact anatomical structure or tissue shown on the screen. This type of “dual recognition” allowed us not only to check the proximal, distal and upper walls of the cavity but to also determine whether the tip of the endoscope had reached the place we were about to curettage, whether there was any corner of the cavity we missed, and what exactly the tissue was that we saw on the screen. It also guides surgeons to the thin area of the cavity bony walls, allowing for the protection of the bone and reducing the risk of pathologic fracture.

NBEE technology also provided an individualized approach to the diagnosis and presurgical planning of patients with large mandibular cystic lesions, which significantly improved the quality of the treatment. The preoperative CT image of lesion segmentation and identification of the surrounding structures and subsequent 3D remodeling allowed the clear display of the topographic features of the involved mandible and the lesion cavity, as well as the 3D location of adjacent mandibular alveolar nerve and impacted teeth. This offered surgeons an opportunity to understand lesions comprehensively to be able to construct a preliminary surgical plan before surgery, including the range of mucoperiosteal incision, position and size of the bony window, and the fixation position and timing of the mandibular DRF. Unlike other reported mandibular navigation cases in which the DRF was fixed on mandibles extraorally [18, 19], in this study, an intraoral DRF was specifically designed for the mandible to avoid extra wounds on the patient’s face. However, the fixation position of the DRF is important, and the position should not only allow the frame and reference markers (light reflecting balls) to be visible in the range of the infrared camera or to interfere with the operation area. With the increase in NBEE cases, we found that the timing of DRF fixation during surgery should also be taken into account. Given the relatively large size of the DRF, if we fix the DRF on the contralateral mandibular alveolar bone at the very beginning of the operation, the patient’s lower lip will be overstretched, leading to swelling of the lip after surgery. Therefore, with the help of presurgical planning, we improved the protocol by fixing the DRF after the mucoperiosteal incision was made, the bony window was exposed and the lesions were removed under endoscopic guidance. We then fixed the DRF and started navigation for precise curettage of lesions involving the ramus, impacted tooth extraction and mandibular alveolar nerve recognition.

## Conclusion

The use of navigation-based endoscopic enucleation (NBEE) for the treatment of large mandibular cystic lesions involving the ramus proved to be an effective method for complete and precise enucleation of the cystic lesion that also preserved the surrounding tissue. It improves the accuracy of surgical manipulations, reduces operational risks, and shortens the operation time and the recovery period. However, further research on this protocol is necessary to maximize accuracy, evaluate efficiency, and assess cost-effectiveness. Instruments that are essential for NBEE should also be improved.

## Data Availability

The datasets used and analysed during the current study available from the corresponding author on reasonable request.

## References

[1] Manor E, Kachko L, Puterman MB (2012). Cystic lesions of the jaws - a clinicopathological study of 322 cases and review of the literature. Int J Med Sci.

[2] McLean AC, Vargas PA (2023). Cystic lesions of the Jaws: The Top 10 Differential diagnoses to ponder. Head Neck Pathol.

[3] Paolo B, Francesco C, Gerardo T (2021). The epidemiology and management of ameloblastomas: a European multicenter study. J Craniomaxillofac Surg.

[4] Paolo B, Francesco C, Anna MA (2022). The epidemiology and management of odontogenic keratocysts (OKCs): a European multicenter study. J Craniomaxillofac Surg.

[5] Ihan, Hren (2008). Miljavec. Spontaneous bone healing of the large bone defects in the mandible. Int J Oral Maxillofac Surg.

[6] Ettl T, Gosau M, Sader R (2012). Jaw cysts - filling or no filling after enucleation? A review. J Craniomaxillofac Surg.

[7] Rubio ED, Mombrú CM (2015). Spontaneous bone Healing after cysts Enucleation without bone grafting materials: a Randomized Clinical Study. Craniomaxillofac Trauma Reconstr.

[8] Wakolbinger R, Beck-Mannagetta J (2016). Long-term results after treatment of extensive odontogenic cysts of the jaws: a review. Clin Oral Investig.

[9] Marin S, Kirnbauer B, Rugani P (2019). The effectiveness of decompression as initial treatment for jaw cysts: a 10-year retrospective study. Med Oral Patol Oral Cir Bucal.

[10] Li X, Zhu K, Liu F (2014). Assessment of quality of life in giant ameloblastoma adolescent patients who have had mandible defects reconstructed with a free fibula flap. World J Surg Oncol.

[11] Gao N, Fu K, Cai JH (2021). [Assessment of the quality of life of mandibular ameloblastoma patients after reconstruction with double-barrel fibula flap]. Zhonghua Er Bi Yan Hou Tou Jing Wai Ke Za Zhi.

[12] Sembronio S, Albiero AM, Zerman N (2009). Endoscopically assisted enucleation and curettage of large mandibular odontogenic keratocyst. Oral Surg Oral Med Oral Pathol Oral Radiol Endod.

[13] Saia G, Fusetti S, Emanuelli E (2012). Intraoral endoscopic enucleation of a solitary bone cyst of the mandibular condyle. Int J Oral Maxillofac Surg.

[14] Huang Z, Huang Z, Zhang D (2016). Endoscopically-assisted operations in the treatment of odontogenic peripheral osteomyelitis of the posterior mandible. Br J Oral Maxillofac Surg.

[15] Romano A, Orabona GD, Abbate V (2016). Endoscope-assisted enucleation of Mandibular Odontogenic Keratocyst tumors. J Craniofac Surg.

[16] Wang Y, Chang S, Lin Z (2020). Endoscopic-assisted enucleation of large mandibular odontogenic cysts. Oral Surg Oral Med Oral Pathol Oral Radiol.

[17] Azarmehr I, Stokbro K, Bell RB (2017). Surgical Navigation: a systematic review of indications, treatments, and outcomes in oral and maxillofacial surgery. J Oral Maxillofac Surg.

[18] de Geer AF, Brouwer de Koning SG, van Alphen MJA (2022). Registration methods for surgical navigation of the mandible: a systematic review. Int J Oral Maxillofac Surg.

[19] Sozzi D, Filippi A, Canzi G (2022). Surgical Navigation in Mandibular Reconstruction: accuracy evaluation of an innovative protocol. J Clin Med.

